# Isolated Upper-Extremity Monoparesis From Basal Ganglia Infarction in a Child With Congenital Internal Carotid and Anterior Cerebral Artery Agenesis: A Case Report

**DOI:** 10.7759/cureus.108496

**Published:** 2026-05-08

**Authors:** Yoseph M Habte, Makida M Habte, Binyam M Habte, Selamawit G Jima, Esimael M Abdu

**Affiliations:** 1 Department of Medicine, Ethio Tebib Hospital, Addis Ababa, ETH; 2 Department of Medicine, Bethel Medical College, Addis Ababa, ETH; 3 Department of Surgery, Debre Berhan University, Debre Berhan, ETH; 4 Department of Surgery, Teklehaimanot General Hospital, Addis Ababa, ETH

**Keywords:** anterior cerebral artery agenesis, basal ganglia infarction, case report, internal carotid artery agenesis, pediatric stroke, pure motor monoparesis

## Abstract

Pediatric stroke is rare and presents with diverse clinical manifestations, often causing delayed recognition and significant long-term morbidity. Isolated upper-extremity monoparesis secondary to basal ganglia infarction in the context of congenital internal carotid artery (ICA) and anterior cerebral artery (ACA) agenesis is extremely uncommon. We report a 14-year-old male presenting with acute left upper-extremity monoparesis. Brain MRI with MRA revealed an acute basal ganglia infarction and congenital agenesis of the right distal ICA and ACA with collateral circulation. Despite well-developed collaterals, ischemia occurred in the lenticulostriate territory, highlighting the vulnerability of subcortical end-arterial regions. The patient had no conventional stroke risk factors, and the subtle neurological deficit underscores the risk of delayed diagnosis. He received antithrombotic therapy, statin therapy, and supportive care, achieving partial neurological recovery. This case highlights the need to consider central causes in children with focal limb weakness and illustrates congenital ICA and ACA agenesis as rare but clinically important contributors to pediatric ischemic stroke.

## Introduction

Pediatric ischemic stroke is an uncommon but potentially devastating neurological condition, with an estimated incidence of 2.3-13 cases per 100,000 children annually, and often presents with atypical clinical features that can delay timely diagnosis [1,2]. Common manifestations include hemiparesis, seizures, and altered mental status, whereas isolated monoparesis is rare, particularly when associated with subcortical basal ganglia infarction [3]. Congenital cerebrovascular anomalies, such as agenesis of the internal carotid artery (ICA) or anterior cerebral artery (ACA), are exceedingly rare but may predispose affected individuals to ischemic events despite the presence of collateral circulation [4,5]. These vascular anomalies can create hemodynamic vulnerabilities in specific cerebral territories, increasing susceptibility to ischemia under otherwise subtle clinical conditions. Early recognition of atypical pediatric stroke presentations is critical, as delayed diagnosis may postpone neuroimaging, secondary prevention, and rehabilitation, potentially worsening long-term neurological outcomes. Here, we report a case of pediatric basal ganglia ischemic stroke in an adolescent presenting with isolated upper-extremity monoparesis in the setting of congenital distal internal carotid and ACA agenesis.

## Case presentation

A 14-year-old right-handed male with no known past medical history or family history presented to the emergency department with a sudden onset of left upper-extremity weakness of 12 hours’ duration. There was no preceding history of head trauma, vigorous physical exertion, excessive crying, laughing episodes, recent infection, or neck manipulation prior to symptom onset. The weakness was not associated with numbness or pain and was more pronounced distally than proximally. According to his mother, he had been unusually sleepy prior to presentation. There was no history of headache, vomiting, fever, neck pain, visual disturbance, seizures, or loss of consciousness. He was not taking any medications and had no history of substance use.

On initial assessment, the patient was conscious and oriented to time, place, and person, with vital signs within normal limits (pulse rate of 72 beats per minute, blood pressure of 100/60 mmHg, respiratory rate of 22 breaths per minute, temperature of 36.5°C, and oxygen saturation of 97% on room air). Neurological examination revealed isolated weakness of the left upper extremity with preserved muscle bulk and normal tone. Muscle strength testing demonstrated proximal weakness graded 4/5 on the Medical Research Council (MRC) scale at the shoulder and elbow, with more pronounced distal weakness involving wrist and finger flexion/extension graded 3/5. Deep tendon reflexes were mildly brisk in the left upper extremity compared with the contralateral side, while plantar responses were flexor bilaterally. Coordination testing was limited by weakness but revealed no definite cerebellar signs. Sensory examination, gait, and cranial nerve function were otherwise normal. The National Institutes of Health Stroke Scale (NIHSS) score was 1. Examination of other systems was unremarkable.

Transthoracic echocardiography and electrocardiography were normal. Due to resource limitations, an extended thrombophilia and autoimmune workup, including protein C, protein S, antithrombin III, antiphospholipid antibodies, factor V Leiden mutation, homocysteine level, and antinuclear antibody testing, could not be performed. However, baseline hematologic and coagulation studies were unremarkable, and there were no clinical features suggestive of systemic autoimmune disease. All other laboratory investigations were within normal limits (Table 1).

**Table 1:** Laboratory investigations with corresponding results and reference values

Laboratory parameter	Result	Normal value
WBC	8.7 × 10^3^/µL	4.0-11.0 × 10^3^/µL
Hemoglobin	14.0 g/dL	13.5-17.5 g/dL
Platelet	342 × 10^3^/µL	150-450 × 10^3^/µL
Lymphocyte percentage	19.1%	15%-50%
Neutrophil percentage	73.5%	45%-80%
Creatinine	0.57 mg/dL	0.6-1.0 mg/dL
Urea	16.3 mg/dL	5-18 mg/dL
Na^+^	142.5 mmol/L	136-145 mmol/L
K^+^	4.41 mmol/L	3.5-5.1 mmol/L
Albumin	4.02 g/dL	3.5-5.2 g/dL
Total protein	6.7 g/dL	6.6-8.3 g/dL
Aspartate transaminase	35.8 U/L	2-50 U/L
Alanine transaminase	20.3 U/L	1-50 U/L
Prothrombin time	14.0 seconds	10.7-14.3 seconds
International normalized ratio	1.17	0.8-1.2
Activated partial thromboplastin time	32.0 seconds	21-35 seconds
Peripheral blood smear	Normal morphology	-

**Figure 1:**
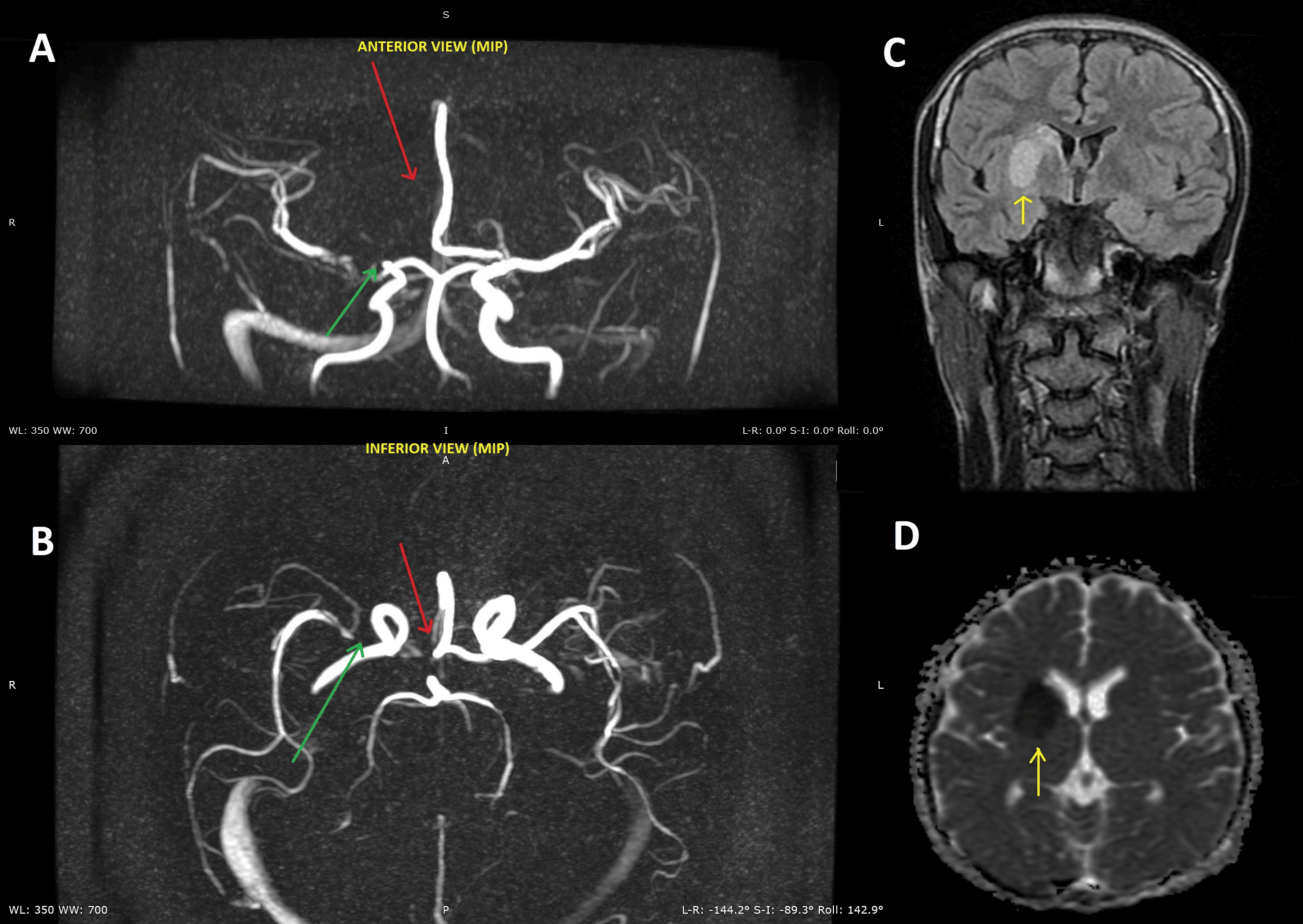
Brain MRI and MRA demonstrating acute basal ganglia infarction with congenital agenesis of the right distal internal carotid artery and right anterior cerebral artery (A, B) Maximum intensity projection (MIP) MRA images in anterior (A) and inferior (B) views demonstrating absent visualization of the right distal internal carotid artery (green arrow) and right anterior cerebral artery (red arrow). (C) Coronal T2-weighted MRI showing a heterogeneously hyperintense lesion in the basal ganglia (yellow arrow). (D) Axial T1-weighted MRI showing a corresponding focal hypointense lesion in the basal ganglia (yellow arrow).

Brain magnetic resonance imaging (MRI) with magnetic resonance angiography (MRA) was performed approximately 13 hours after symptom onset and demonstrated an acute basal ganglia infarction, appearing as a focal T1-weighted hypointense and T2-weighted heterogeneously hyperintense lesion with diffusion restriction and no evidence of hemorrhage. The MRA showed absent visualization of the right distal ICA and right ACA, narrowing of the proximal (M1) segment of the right middle cerebral artery, and enlarged transcranial collateral vessels arising from the external carotid artery system, with compensatory enlargement of the left ACA. These findings were suggestive of congenital agenesis of the right distal ICA and right ACA with collateral circulation (Figure 1). The remainder of the intracranial arterial and venous vasculature, as well as the cervical spine MRI, were unremarkable. Congenital agenesis was favored over chronic occlusion due to the absence of an abrupt arterial cutoff or residual stenotic segments, together with well-developed collateral circulation and compensatory enlargement of contralateral vessels, collectively suggesting a longstanding developmental vascular anomaly rather than an acquired occlusive process.

The patient was diagnosed with acute ischemic stroke involving the basal ganglia, presenting as isolated left upper-extremity monoparesis in the setting of suspected congenital cerebrovascular anomalies. Shortly after exclusion of intracranial hemorrhage, antithrombotic therapy was initiated with aspirin and short-term clopidogrel, along with atorvastatin, intravenous fluids, gastroprotective therapy, and supportive care.

During hospitalization, the patient remained hemodynamically and neurologically stable, with gradual improvement in motor strength. At discharge after two days, mild residual left upper-extremity weakness persisted (MRC grade 4/5). He was referred for physiotherapy and scheduled for close outpatient neurology follow-up. Long-term neurological outcome and risk of recurrence remain to be determined.

## Discussion

Pediatric ischemic stroke is an uncommon but serious neurological condition associated with substantial long-term morbidity. Its clinical presentation is often variable and subtle, contributing to delayed recognition and management. While hemiparesis, seizures, and altered mental status are typical presentations, isolated monoparesis is rare and may further obscure timely diagnosis [6-9].

This case illustrates an unusual presentation of pediatric acute ischemic stroke as isolated upper-extremity monoparesis due to basal ganglia infarction in the setting of congenital distal ICA and ACA agenesis. To our knowledge, this combination of basal ganglia infarction presenting as isolated upper-extremity pure motor monoparesis in association with distal ICA and ACA agenesis has not previously been reported in the pediatric population, although atypical or subtle pediatric stroke presentations may be underrecognized and underreported. The occurrence of ischemia within the lenticulostriate territory despite well-developed collateral circulation highlights the susceptibility of end-arterial regions, which lack sufficient compensatory perfusion under altered hemodynamic conditions.

Pure motor monoparesis is an uncommon stroke presentation, reported in 2% to 13% of stroke patients, and more frequently associated with cortical infarctions involving the precentral gyrus or superficial middle cerebral artery territories [3]. In contrast, subcortical lesions are less commonly implicated, particularly in children. The present case demonstrates that basal ganglia infarction can produce a focal motor deficit in isolation, reinforcing the need to consider central etiologies even in the absence of sensory involvement or cortical signs.

Congenital agenesis of the ICA is an exceptionally rare vascular anomaly, with an estimated incidence of less than 0.01% [4,5]. It is thought to result from disruption of embryologic vascular development [4] and is often clinically silent due to compensatory collateral circulation [10]. However, ischemic events may occur when these compensatory pathways are insufficient to maintain adequate perfusion in vulnerable territories [11]. ICA agenesis can be classified into six main types, with distal or terminal segment agenesis corresponding to Type B [5]. In this patient, imaging demonstrated distal (Type B) ICA agenesis with collateral supply from the contralateral ACA and external carotid system. Despite these adaptations, infarction occurred within the basal ganglia, underscoring the limitations of collateral circulation in end-arterial regions. Unilateral moyamoya disease was considered as a differential diagnosis; however, the absence of progressive steno-occlusive changes and imaging features favoring developmental vascular absence supported congenital agenesis rather than moyamoya vasculopathy.

The management of pediatric acute ischemic stroke remains challenging due to limited evidence from randomized controlled trials [8,9]. MRI with diffusion-weighted imaging is the diagnostic modality of choice, allowing early detection and differentiation from stroke mimics [9,12]. In this case, early MRI and MRA were critical in confirming the diagnosis and identifying the underlying vascular anomaly despite a mild clinical presentation.

Antithrombotic therapy remains the cornerstone of management following exclusion of intracranial hemorrhage, particularly in patients without arterial dissection, cardioembolic sources, or known hypercoagulable disorders [8,9]. In this patient, dual antiplatelet therapy was initiated in the acute phase, given the presumed arterial etiology, with plans for reassessment during follow-up. Reperfusion therapies, such as intravenous thrombolysis or mechanical thrombectomy, are generally reserved for carefully selected pediatric patients [13]; however, delayed presentation, mild neurological deficit, and absence of large-vessel occlusion precluded their use in this case.

This report has limitations. Comprehensive evaluation for underlying prothrombotic conditions was not performed due to resource constraints, which may limit full etiological assessment. Additionally, long-term follow-up data are not yet available.

In summary, this case emphasizes that pediatric ischemic stroke may present with subtle and atypical deficits, such as isolated monoparesis, and highlights congenital ICA agenesis as a rare but important underlying etiology. Early neuroimaging and careful vascular evaluation are essential to ensure timely diagnosis and appropriate management.

## Conclusions

Pediatric ischemic stroke can present with subtle and atypical deficits, such as isolated upper-extremity monoparesis, which may delay recognition. Congenital cerebrovascular anomalies, including agenesis of the internal carotid and anterior cerebral arteries, are rare but can predispose children to ischemia despite the presence of collateral circulation. This case highlights the importance of maintaining a high index of suspicion, performing timely neuroimaging, and providing individualized management to optimize outcomes. Early identification of rare vascular substrates is essential for guiding follow-up, prognosis, and secondary prevention in pediatric stroke patients.
